# The Efficacy and Cost-Effectiveness of Stepped Care Prevention and Treatment for Depressive and/or Anxiety Disorders: A Systematic Review and Meta-Analysis

**DOI:** 10.1038/srep29281

**Published:** 2016-07-05

**Authors:** Fiona Yan-Yee Ho, Wing-Fai Yeung, Tommy Ho-Yee Ng, Christian S. Chan

**Affiliations:** 1 Department of Psychology, The University of Hong Kong, Hong Kong; 2 School of Nursing, The Hong Kong Polytechnic University, Hong Kong; 3 Department of Psychology, Temple University, United States

## Abstract

Stepped care is an increasingly popular treatment model for common mental health disorders, given the large discrepancy between the demand and supply of healthcare service available. In this review, we aim to compare the efficacy and cost-effectiveness of stepped care prevention and treatment with care-as-usual (CAU) or waiting-list control for depressive and/or anxiety disorders. 5 databases were utilized from its earliest available records up until April 2015. 10 randomized controlled trials were included in this review, of which 6 examined stepped care prevention and 4 examined stepped care treatment, specifically including ones regarding depressive and/or anxiety disorders. Only trials with self-help as a treatment component were included. Results showed stepped care treatment revealed a significantly better performance than CAU in reducing anxiety symptoms, and the treatment response rate of anxiety disorders was significantly higher in stepped care treatment than in CAU. No significant difference was found between stepped care prevention/treatment and CAU in preventing anxiety and/or depressive disorders and improving depressive symptoms. In conclusion, stepped care model appeared to be better than CAU in treating anxiety disorders. The model has the potential to reduce the burden on existing resources in mental health and increase the reach and availability of service.

Depressive and anxiety disorders bring severe health and financial burdens to the sufferers and the public^1^,^2^. Although extensive evidence has shown that psychological treatment is effective in treating depressive and anxiety disorders^3^,^4^,^5^, resource constraints limit its reach, especially in countries with developing economies. Per 100,000 people in low-income countries, there are merely 5% and 4% of them that are psychiatrists and psychologists respectively^6^. Given the significant discrepancies between the demand for evidence-based psychotherapy and the availability of healthcare professionals^7^, it is crucial to make efficient use of the limited healthcare resources to maximize the number of beneficiaries.

Recently, stepped care model is considered as one of the possible solutions to reduce the existing healthcare burden^8^. Within the stepped care model (Supplementary Fig. S1), evidence-based psychological treatments are distributed to different steps^9^. This starts with less intensive treatments, for example, through self-help treatments delivered through the internet^10^, bibliotherapy^11^, and/or group therapy^12^, to more intensive treatments involving individual therapy provided by specialists^13^ and possibly subsequent pharmacological treatment^14^. Stepped care model as a healthcare delivery method has two core features, “least restrictive” and “self-correcting”^15^. “Least restrictive” refers to a low-intensity, cost effective, and least time consuming feature of this method and is used as the first-line treatment. “Self-correcting” refers to the “stepping-up” criteria that are utilized in possible preparation of more intensive and expensive treatment, and this is necessary based on treatment outcome. Patients are monitored systematically and referred to the next step if they do not respond significantly to the prior steps in the model. A care manager or psychiatric nurse is sometimes assigned to coordinate the treatment program, monitor the progress, and assist patients to decide the level of treatment, all of which take into account of the severity of their symptoms^16^. The advantage of the stepped care model is that it maximizes the effectiveness and efficiency of treatments by optimizing resource allocation.

Currently, stepped care model is recommended in the clinical guidelines by the National Institute for Health and Clinical Excellence in the United Kingdom for common mental health problems^8^. The model has been implemented and evaluated for various mental health problems such as eating disorders^17^, depression and anxiety^18^, obsessive-compulsive disorder^19^, posttraumatic stress disorder^20^, chronic fatigue syndrome^21^, nicotine dependence^22^, and alcohol use disorders^23^. The findings of some of these randomized controlled trials (RCTs) support the clinical benefits of stepped care approach. For example, stepped care treatment was found superior than CAU in treating eating disorders in a 1-year follow-up^17^ and alcohol use disorders in a 6-month follow-up^23^. On the other hand, other clinical trials failed to show significant outcome differences between the stepped care and CAU groups^18^. Nonetheless, the stepped care model appears to be more cost effective than traditional approaches. For example, stepped care was more cost effective in comparison to traditional cognitive-behavioral therapy (CBT) in treating bulimia nervosa^24^. The cost per abstinent subject of stepped care and traditional CBT were USD $12,146 and USD $20,317 respectively.

Given the potential benefits of the stepped care model on the one hand, and the structural constraints of traditional modes of treatment delivery on the other, it would be of interest to systematically examine its clinical efficacy and cost effectiveness. To our knowledge, there are only two systematic reviews that summarized the efficacy of the stepped care model; one for alcohol use disorders and nicotine dependence^25^ and the other for depression^26^. The former found little evidence to conclude that the stepped care model is superior to CAU in terms of treatment outcome but they recognized that this might be partially due to insufficient statistical power. The latter, on the other hand, found moderate effect size in treating depression, but had limited evidence to conclude that stepped care should be the dominant treatment model. The review by van Straten^26^ has summarized the studies on stepped care model as a treatment intervention for depression, but the review did not include studies on stepped care model as a preventive intervention, which has important clinical and public health implication.

There is great heterogeneity in the architecture of stepped care model, in terms of the number of steps and treatment components incorporated, which may limit the generalizability of the results in previous meta-analysis^26^. We argue that self-help is a crucial first treatment step in the model because of its cost effectiveness and least restrictive nature. The inclusion of self-help treatment has the advantage of allowing a larger proportion of patients to reach the service, especially for individuals who are not able to afford pharmacotherapy or psychotherapy and/or those who have geographical or transportation constraints^27^,^28^. However, to date, there is no systematic review and meta-analysis of stepped care prevention and treatment for depressive and anxiety disorders that specifically investigated studies using self-help treatment as the first step in stepped care model. Given these knowledge gaps, we conducted a meta-analysis of RCTs that examined the efficacy of stepped care vis-à-vis CAU or waiting-list (WL) as a treatment or prevention intervention for depression or/and anxiety. We included both prevention and intervention studies. Furthermore, cost-effectiveness of stepped care approach was also evaluated.

## Methods

### Selection of Studies

Two authors (FYH and THN) independently searched electronic databases, including Cochrane Central Register of Controlled Trials, PubMed, PsycINFO, CINAHL Plus, and ProQuest Dissertations & Theses from the earliest available records to April 2015. The search terms were indicative of stepped care treatment and prevention and RCTs: (psychotherapy OR psychological treatment OR cognitive-behavioral OR CBT OR mental OR counsel*) AND (random* OR controlled trial OR randomized controlled trial OR RCT) AND (stepped care OR adjunctive treatment OR treatment tiering OR adaptive treatment). The combination of terms was searched by title, abstract or keyword. We also searched for additional relevant articles from the reference lists of retrieved papers. We did not set any restrictions for duration of treatment, outcome measure or study quality.

Studies that employed stepped care model as a treatment or prevention intervention for depressive and/or anxiety disorders were included in this review. Prevention referred to intervention provided to people with high risk or subthreshold symptoms of depressive and anxiety disorders; whereas treatment referred to intervention given to those with a diagnosis of a depressive and/or anxiety disorder. Our inclusion criteria were the following: (1) studies examining stepped care treatment or prevention in comparison with CAU or WL; (2) participants with depressive and/or anxiety disorders diagnosed by standard diagnostic criteria such as Diagnostic and Statistical Manual of Mental Disorders (DSM-IV or DSM-5) or chief complaint of depressive and/or anxiety symptoms assessed by standardized measures; and (3) studies with self-help as the first treatment step in stepped care model (i.e., “least restrictive”) without a combination of non-self-help treatments, because self-help was considered appropriate to be the first line treatment due to its low cost and wide availability^27^,^29^. We excluded studies with no “stepping-up” criteria (i.e., “self-correcting”). Collaborative care RCTs were considered as eligible if both the inclusion and exclusion criteria were met.

### Data Extraction

Data extraction was performed by two authors (FYH and THN) independently. Disagreements on inclusion and exclusion were resolved through discussion. Consensus was achieved for all included publications. The methodological qualities in the included studies were evaluated by the Cochrane’s risks of bias assessment^29^. The risks-of-bias assessment covers six domains in evaluating RCTs, including selection bias, performance bias, detection bias, attrition bias, reporting bias, and other possible biases. Assessors assigned a judgment for each domain, which can be “yes” (low risk of bias), “no” (high risk of bias) or “unclear” (uncertain risk). An inter-rater reliability analysis using the Kappa statistic was performed on the quality assessment, and the agreement was substantial (Cohen’s Kappa = 0.76, *p* < 0.001). Discrepancies were resolved by discussion with the third author (WFY).

### Statistical Analyses

The program Review Manager (RevMan 5.2.4) was used for statistical analysis. Continuous outcome data were combined using either mean difference or standardized mean difference with 95% confidence intervals (CI); dichotomous outcome data were combined using risk ratio and odds ratio with 95% CI. Random effects model was used in view of the anticipated between-study variance^29^. Publication bias was evaluated by funnel plot if 10 or more studies were included^29^.

## Results

### Selection of Studies

**Figure 1:**
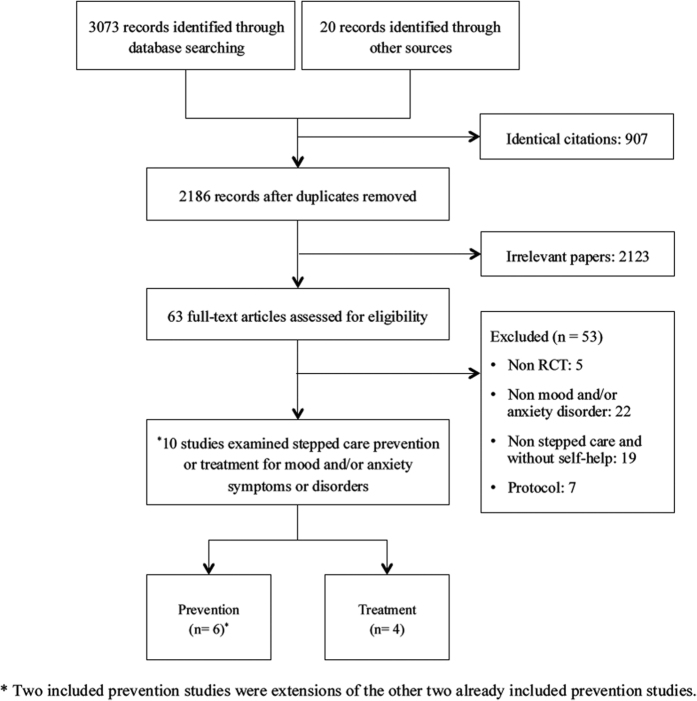
Selection Flow of Trials for Inclusion in the Review.

There were 3093 citations identified from the search, of which 907 were identical citations and 2123 were irrelevant papers. A total of 63 full texts were retrieved for further review, of which 53 articles were excluded due to the violation of inclusion criteria (Fig. 1). Of the remaining 10 RCTs included in this review, 6 examined stepped care prevention and 4 examined stepped care treatment. Since 2 included prevention studies were extensions of the other 2 already included prevention studies, the total number of included studies were greater than the actual number of trials described in the tables and figures. Details of the excluded studies are available from the authors.

### Description of Included Studies

#### Stepped care prevention

The four stepped care prevention trials^30^,^31^,^32^,^33^,^34^,^35^ included a total of 731 participants (sample size ranged from 136 to 240) (Table 1). Participants were elderly from the Netherlands with depressive or anxiety symptoms and adults from Hong Kong with subthreshold depression and/or anxiety. Participants’ mean age was 78.2 years and 68.6% of the sample was female. Standardized self-rating scales, the Center for Epidemiologic Studies Depression Scale (CES-D) and the Hospital Anxiety and Depression Scale–Anxiety (HADS-A) were used in three and one of the included trials for screening respectively; however, a different cutoff score was adopted. The remaining trial was a relapse prevention, which only included participants who had undergone previous psychological or pharmacological treatment for depression (Study 1)^30^,^31^. The major inclusion and exclusion criteria are presented in Supplementary Table S1. All studies compared stepped care prevention with CAU. Participants had unrestricted access to any form of health care (e.g., psychological interventions and prescription medications) in CAU group. Treatment outcome was evaluated with self-report measures in all four studies and with structured diagnostic interviews in three studies.

#### Stepped care treatment

The four stepped care treatment trials included a total of 488 participants; the sample size ranged from 30 to 180 (Table 1)^16^,^18^,^19^,^36^. The average age of the participants was 43.9 years and 63.5% of the sample was female. Participants were adults aged ≥18 years from the Netherlands or United States with depressive and/or anxiety disorders. The Diagnostic and Statistical Manual of Mental Disorders, Fourth Edition (DSM-IV) was used in all trials for screening. All studies compared stepped care treatment with CAU. Participants had unrestricted access to any form of health care (e.g., no treatment, psychological interventions, prescription medications, referrals to psychiatric nurse, primary care psychologist, specialized mental health center or other professional) or standard exposure and response prevention (ERP)^19^ in CAU group. Treatment outcome was evaluated with self-rated or clinician-administered questionnaires.

### Description of Stepped Care Content

#### Stepped care prevention

In the identified stepped care prevention studies, all of the prevention programs consisted of 4 steps, with watchful waiting as the first step, self-help psychotherapy as the second step, face-to-face psychotherapy as the third step and referral to specialists as the last step. Self-help psychological treatments were based on CBT from the book *Coping with Depression*^37^ in all except one trial (Study 4)^35^. Various treatment contents were adopted in face-to-face psychotherapy step, including CBT, life review therapy and problem-solving treatment (PST) (Table 2).

#### Stepped care treatment

In the identified stepped care treatment studies, the treatment program consisted of 2 to 4 steps. One study used 4 steps with the same treatment sequence as stated in stepped care prevention; one study used 3 steps with self-help psychotherapy as the first step, followed by face-to-face psychotherapy and pharmacotherapy; two studies used 2 steps with self-help and face-to-face psychotherapy. Self-help psychological treatments included PST, exposure therapy, ERP, and CBT. Face-to-face psychological treatments were based on PST, CBT and ERP. Details of the stepped care treatment content are described in Table 2.

### “Stepping Up” Criteria

#### Stepped care prevention

“Stepping up” refers to the progression from less restrictive treatments to more intensive treatments if no significant health benefits were achieved. All of the four prevention trials invited participants to progress to the next step if the symptom severity, as measured by CES-D and HADS-A, remained greater than a cutoff score (Table 2). Different cutoff scores were used across four studies: They were CES-D score ≥15, ≥16, less than a 5-point improvement or HADS-A ≥6, respectively. Direct referral to the last step was given to participants who had a diagnosis of depressive and/or anxiety disorders in three studies.

#### Stepped care treatment

The stepping-up criteria were diverse across the four included treatment studies (Table 2). One of the studies^18^ adopted stringent criteria, with a combination of three self-report assessments, including Inventory of Depressive Symptomatology (IDS) ≥14, Hospital Anxiety and Depression Scale (HADS) ≥8, and Work and Social Adjustment Scale (WSAS) ≥6. The other 3 studies used Beck Anxiety Inventory (BAI) >11 or less than 50% score reduction^16^, Clinical Global Impression-Severity Scale (CGI-S) ≥3^36^, or the Yale–Brown Obsessive–Compulsive Scale (Y-BOCS) >13^19^. Serious cases were referred to the last step if the following criteria were fulfilled: WSAS ≥8 on 3 of the 4 daily functioning domains, depression with psychotic features or suicidal ideation, or Y-BOCS >13. One of the included studies did not report the direct referral requirement (Study 4)^16^.

### Attrition Rate and Reasons for Attrition

#### Stepped care prevention

Attrition rate was reported in all included prevention studies; however, the assessment time points were different across studies (Table 2). Three of the four studies reported the attrition rates in both groups. The average cumulative attrition rates of stepped care prevention were 15.0% at posttreatment, and the corresponding rate of CAU was only reported in 3 included studies, which was 8.9% at posttreatment. There was significant difference between stepped care prevention group and CAU group in attrition rate (risk ratio: 1.7, 95% CI: 1.2 to 2.4, *p* < 0.01 *I*^*2*^ = 40%, *N* = 3). Reasons for attrition were reported in all four studies. The most commonly cited reasons were physical illness and death, which might be due to the advanced age of the target population.

#### Stepped care treatment

Attrition rate was reported in three of the four included treatment studies (Table 2). Of the three treatment studies, two reported the attrition rates of both stepped care and CAU groups. The average cumulative attrition rates of stepped care treatment were 19.4% at posttreatment, and the corresponding rates of CAU were 5.0%. No significant difference was found between stepped care treatment group and CAU group in attrition rate (risk ratio: 1.9, 95% CI: 0.9 to 4.3, *p* = 0.11, *I*^*2*^ = 0%, *N* = 2). The dropouts provided various attrition reasons including unwillingness to complete assessment, contact failure, and physical illness.

### Quality Assessment

#### Cochrane’s risks of bias assessment

There were some methodological flaws in all of the included studies (Supplementary Table S2). Due to the nature of the study, it made blinding of participants improbable, and thus the risk of bias was assessed as “high” in all included studies. On the other hand, because the independent assessor was usually masked from the conditions, the risk of bias for blinding of assessor was generally assessed as “low.” The majority of studies did not report the allocation concealment method and thus the risk of bias were rated as “uncertain.” In addition, all included studies were free of bias in selective reporting and other possible biases.

### Meta-analyses

#### Stepped care prevention vs. CAU

**Figure 2:**
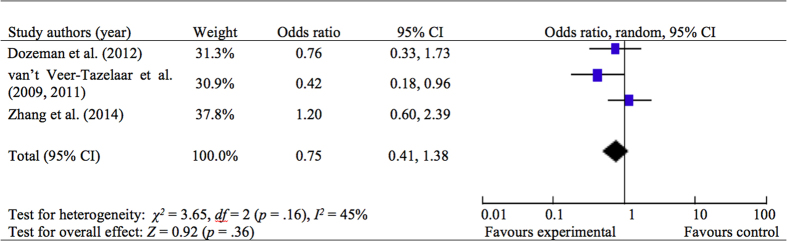
Stepped Care Treatment vs. CAU control on the Incidence of Anxiety and/or Depressive Disorders at Immediate Posttreatment.

The incidence rate of anxiety and/or depressive disorders in stepped care prevention did not differ significantly from CAU (Fig. 2; OR = 0.75, 95% CI: 0.41, 1.38, *p* = 0.36, *I*^*2*^ = 45%, *N* = 3), indicating that the chances of developing anxiety and/or depressive disorders in stepped care group and CAU group were similar.

#### Stepped care treatment vs. CAU

**Figure 3:**
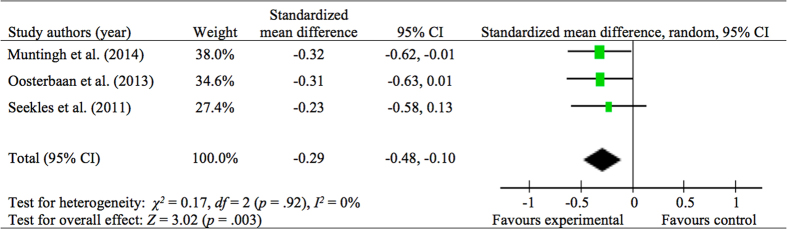
Stepped Care Treatment vs. CAU control on Anxiety Symptoms at Immediate Posttreatment.

At immediate posttreatment, the pooled analysis of three RCTs found that stepped care treatment was significantly better than CAU in reducing anxiety symptoms (Fig. 3; standardized mean difference = −0.29, 95% CI: −0.48, −0.10, *p* < 0.01, *I*^*2*^ = 0%). The pooled treatment response rate of anxiety disorders in two RCTs was significantly higher in stepped care treatment than in CAU (Supplementary Fig. S2; OR = 2.38, 95% CI: 1.25, 4.52, *p* < 0.01, *I*^*2*^ = 0%). No significant difference was found between stepped care treatment and CAU in improving depressive symptoms.

### Cost-effectiveness

Among the included stepped care prevention studies, two reported data on cost-effectiveness (Study 2 and 3)^38^,^39^. Both studies evaluated stepped care prevention in the older population. In Study 2, the cost estimate was derived from Trimbos and Institute of Medical Technology Assessment Questionnaire (TIC-P) at each prevention step^38^. It demonstrated that there was no significant difference in total mean cost between stepped care prevention (€4,284) and CAU (€3,446), and the prevention was not considered cost-effective when comparing with CAU in elderly. Study 3 found that stepped care prevention halved the incidence rate of depression and anxiety at an incremental cost of €563 per recipient and an average of €4,367 for a depression/anxiety-free year^39^. The cost was estimated based on the healthcare uptake measured by the TIC-P and out-of-pocket expenses from patients at each prevention step. The study concluded that the stepped care prevention allowed depression/anxiety-free survival years in elderly at an affordable cost. Data on productivity cost was considered irrelevant in the older population. Hence, it was not included in the cost-effectiveness analysis in both studies. Cost-effectiveness was not examined in any of the treatment studies that were included.

### Publication Bias

Funnel plots for the comparison of stepped care prevention or treatment and CAU were not possible due to the small number of included studies; hence, publication bias could not be determined.

## Discussion

Stepped care model is an emerging delivery method in preventing and treating depression and anxiety disorders. The aim of this review study was to compare the efficacy and cost-effectiveness between stepped care and CAU groups. The comparison of stepped care treatment and CAU revealed significant difference in favor of the former in terms of anxiety symptoms and treatment response rate of anxiety disorders. Significant difference was not found between stepped care prevention/treatment and CAU in preventing anxiety and/or depressive disorders and reducing depressive symptoms. Cost-effectiveness analysis demonstrated inconsistent findings between stepped care prevention and CAU in two studies, with one considered effective at an affordable cost but not the other. Overall, there was evidence showing that stepped care treatment is effective in alleviating anxiety symptoms.

In our meta-analysis, sample sizes merged from multiple studies increased the chance of detecting the difference of treatment effect between the two groups, if any. On the other hand, stepped care prevention had significantly higher attrition rate than CAU, perhaps patients in the stepped care group lost their interest and patience to step up. The included studies had various treatment components at each step and a wide range of stepping-up criteria, which may have affected the treatment effect of the whole treatment model. Nonetheless, the heterogeneity test in the meta-analysis of stepped care treatment indicated no observed statistical heterogeneity (i.e., *I*^*2*^ = 0%); therefore, the detected treatment effect was likely to be genuine, given the high level of consistency across the studies^40^.

To date, there is no consensus on the structure of the stepped care model. Indeed, other than the fact that they all included self-help as a treatment component, the RCTs included in the current review have different combinations, sequences, and number of steps in their rendition of the model. Although there might be merit to standardize the architecture of the model, we argue that flexibility is also needed to adequately respond to the differences in resource availability and logistical constraints across diverse settings. As such, stepped care model, we argue, should be evaluated as a whole, and not independently at each step. Researchers are encouraged to focus their attention on constructing the most feasible model in response to the needs and available resources of the particular setting. Nevertheless, there remains a need to select evidence-based treatments as the basis of the model, such as, but not limited to, CBT^41^,^42^, PST^43^, behavioral activation^44^, and mindfulness and acceptance-based treatments^45^. Generally, the delivery modality of these evidence-based treatments comprises self-help therapy, guided group therapy, brief and long-term individual therapy. In terms of number of steps in the model, the prevention studies we included generally had four steps, whereas treatment studies adopted two to four steps. The optimal number of steps can vary under different circumstances. Two major factors—the intensity of treatment and the upper limit of therapist input in a routine healthcare setting—can help to determine how many steps should be included^15^.

Self-correcting is one of the key features on the health gain and patients’ progress^15^. It is underscored that whether or not a model is cost-effective may partly depend on the stepping-up criteria^8^. A well-defined stepping-up criterion should be sensitive enough to detect those who fail to respond to the first-line treatments, so that the model is able to maximize the proportion of patients who may benefit from the low-intensity first-line interventions. On the one hand, it should be able to rapidly identify and provide appropriate treatment to patients in need of more intensive care.

The heterogeneity of the stepping-up criteria among the included studies did not allow us to draw a conclusion on a best-fit assessment tool or cut-off score. Conventional questionnaires for screening depression and/or anxiety, such as CES-D^46^, BDI^47^, BAI^48^, and HADS^49^, can be considered as screening tools. The cut-off score can be decided based on previous validation studies with good sensitivity and specificity analyses. Nonetheless, there is room for cut-off score adjustment depending on the nature of trial. For example, relative to treatment studies, prevention studies tend to recruit high-risk populations who do not meet full criteria for a diagnosis. For this reason, prevention studies might have relatively more conservative cut-off scores whereas treatment studies might adopt more stringent cut-off scores. Alternatively, the option for clinician administrated structured diagnostic assessments is more desirable. Nonetheless, the additional therapist time and cost may undermine the core feature (i.e. least restrictive) of the stepped care model.

There were several limitations in our study. First, the number of studies included in the meta-analysis was small. Since stepped care for depression and anxiety is a relatively new area of research, only few trial studies have been completed. The exclusion of non-self-help treatment at the entry step also reduced the amount of studies. Nevertheless, since the step care model varied greatly across studies, the inclusion of stepped care studies regardless of their model design may hinder the comparison between studies. We encourage researchers to interpret the current findings with caution, and extend and replicate the current findings in the future to provide further evaluations for stepped care.

Second, the variation of treatment effect among studies might be related to different model structures. The content, duration, sequence and delivery method of treatment might have an impact on the treatment outcome. In addition, the diversity of diagnostic criteria and age groups in the included studies may also contribute to the variation in the treatment outcome. Finally, most of the included studies were from healthcare systems in European or American contexts, with the majority of trials conducted in the Netherlands. It is unknown whether the stepped care model is applicable or generalizable to healthcare systems in emerging countries where resources for mental health might be scarce. To date, no studies examining the efficacy of stepped care for depressive and anxiety disorders have been conducted in emerging countries with very limited mental healthcare resources.

In response to the high demand yet simultaneously limited resources for mental health services, the stepped care model for common mental health disorders is of increasing relevance around the globe. The result in this review study sheds light on the efficacy and cost-effectiveness of stepped care model in preventing and treating depressive and/or anxiety disorders. The model has the potential to reduce the burden on existing resources in mental health and at the same time increase the reach and availability of service. The model might also be of particular interest in regions where stigma is preventing those in need of mental health services to seek help. The lower steps in the stepped care model may be less stigmatizing than full-blown psychotherapy or psychiatric care. We suggest further fine-tuning of the stepped care model with the utilization of technology to further enhance its efficacy and efficiency. A smartphone application, for example, might be a favourable platform to support patients in clinical settings^50^. It can even serve as a tool used in the first step (e.g., self-help). Emerging countries have become more aware of the importance of mental health service. Unfortunately, investment on resources continues to be lacking^51^. Stepped care model serves as an economically viable and effective treatment option.

## Additional Information

**How to cite this article**: Ho, F. Y.-Y. *et al*. The Efficacy and Cost-Effectiveness of Stepped Care Prevention and Treatment for Depressive and/or Anxiety Disorders: A Systematic Review and Meta-Analysis. *Sci. Rep*. **6**, 29281; doi: 10.1038/srep29281 (2016).

## Supplementary Material

Supplementary Information

## Figures and Tables

**Table 1:** Characteristics of Randomized Controlled Trials of Stepped Care Treatment and Prevention for Depressive and/or Anxiety Disorders.

No.	Study authors (year)	Country/type of participants	Mean age (*SD*)/% female	Diagnostic criteria	Design	Collaboration	Sample size (subgroup)	Assess-ments	Stepped care intervention	Control intervention	Outcome measure	Major results reported
**Stepped Care Prevention**												
1	Apil *et al*.^30^,^31^	Netherlands**/**elderly with depression previously	65.8 (8.4)**/**73.2%	Had received treatment for depression	2-parallel arms (SC, CAU)	Nurses	136 (74/62)	6, 12, 24 mo	1) Watchful waiting; 2) SH CBT; 3) FTF CBT; 4) referral to physicians or psychotherapists	CAU (unrestricted access to any form of health care)	CES-D, GGZ, Tic-P	No significant difference between SC and CAU in incidence of depression at 12-mo. SC required new treatment significantly>CAU.
2	Dozeman *et al*.^32^	Netherlands**/**elderly in residential homes	84.4 (6.6)**/**72.9%	CES-D ≥8, MINI for depressive or anxiety disorders	2-parallel arms (SC, CAU)	General practitioners, mental health specialists, nurses	185 (93/92)	1, 4, 7, 10 mo	1) Watchful waiting; 2) SH activity-scheduling; 3) FTF life review; 4) referral to general practitioners or mental health specialists	CAU (unrestricted access to any form of health care)	MINI, CES-D, HADS-A, loneliness scale, Tic-P, ADL, GARS	SC significantly reduced the risk of MDD incidence in comparison with CAU.
3	van’t Veer-Tazelaar *et al*.^33^,^34^	Netherlands**/**elderly with subthreshold depression and anxiety	81.4 (3.7)**/**74%	CES-D ≥16, MINI for depressive or anxiety disorders	2-parallel arms (SC, CAU)	Nurses, primary care physicians	170 (86/84)	6, 12, 18, 24 mo	1) Watchful waiting; 2) SH CBT; 3) FTF PST; 4) referral to primary care physicians	CAU (unrestricted access to any form of health care)	CES-D, MINI	SC significantly halved the cumulative incidence rate of DSM-IV depression or anxiety at 12 and 24-mo FU.
4	Zhang *et al*.^35^	Hong Kong**/**adults in public primary clinics	NA (NA)**/**74.2%	CES-D ≥16 or HADS-A ≥6	2-parallel arms (SC, CAU)	Social workers, family medicine doctors	240 (121/119)	3, 6, 9, 12, 15 mo	1) Watchful waiting; 2) guided SH; 3) problem solving treatment; 4) family doctor treatment	CAU (unrestricted access to any form of health care)	CES-D, HADS-A	No significant difference between SC and CAU in of SCP in preventing the onset of MDD and GAD.
**Stepped Care Treatment**												
5	Muntingh *et al*.^16^	Netherlands**/**adults in primary care	46.5 (15.5)**/**68.3%	DSM-IV for PD and GAD	2-parallel arms (SC, CAU)	Care managers, general practitioners, psychiatrists	180 (114/66)	3, 6, 9, 12 mo	1) Guided SH CBT; 2) FTF CBT; 3) pharmacotherapy	CAU (unrestricted access to any form of health care)	BAI, PHQ, SF-36, EQ-5D	SC significantly gained more in BAI score than CAU.
6	Oosterbaan *et al*.^36^	Netherlands**/**outpatients in mental healthcare centres	38.0 (12.0)**/**62.0%	MINI for depressive, anxiety and stress-related disorders	2-parallel arms (SC, CAU)	General practitioners, psychologists, nurses	158 (94/64)	4, 8, 12 mo	1) Guided SH CBT in primary care; 2) FTF CBT in mental healthcare; 3) intensive psychiatric treatment in day care clinic	CAU (unrestricted access to any form of health care)	CGI-I, CGI-S, HRSA, CES-D, FQ, SCL-90-R, SF-36	SC significantly superior to CAU responders at 4-mo mid-test. No significant difference between SC and CAU at 8 and 12-mo FU.
7	Seekles *et al*.^18^	Netherlands**/**primary care	50.2 (11.2)**/**65%	DSM-IV for depressive and/or anxiety disorders**/**HADS ≥12	2-parallel arms (SC, CAU)	General practitioners, psycholo-gists, psychiatric nurses	120 (60/60)	8, 16, 24 wk	1) Watchful waiting; 2) guided SH CBT; 3) FTF PST; 4) referral**/**pharmacotherapy	CAU (unrestricted access to any form of health care)	IDS, HADS, WSAS, CIDI	Both groups significantly decreased in depression and anxiety over time. No significant difference between SC and CAU in depression and anxiety.
8	Tolin *et al*.^19^	NR	33.9 (13.3)**/**58.8%	DSM-IV for OCD ≥1 year, Y-BOCS ≥16 and CGI ≥4	2-parallel arms (SC, CAU)	Therapists	30 (18/12)	1, 3 mo	1) Guided SH ERP; 2) FTF ERP	Standard ERP	Y-BOCS, CGI-S and CGI-I	No significant difference between SC and CAU in Y-BOCS and treatment satisfaction.

ADL, activity of daily living; BAI, Beck Anxiety Inventory; CAU, care-as-usual; CBT, cognitive-behavioral therapy; CES-D, Centre of Epidemiological Studies–Depression scale; CGI-I, Clinical Global Impression–Improvement Scale; CGI-S, Clinical Global Impression–Severity Scale; CIDI, Composite International Diagnostic Interview; DSM-IV, Diagnostic and Statistical Manual of Mental Disorders, Fourth Edition; ERP, exposure and response prevention; EQ-5D, EuroQol-5D; FQ, Fear Questionnaire; FTF, face-to-face; FU, follow-up; GAD, generalized anxiety disorder; GARS, Groningen Activity Restriction Scale; GGZ, GGZ thermometer; HADS-A, Hospital Anxiety and Depression Scale–Anxiety; HRSA, Hamilton Rating Scale for Anxiety; IDS, Inventory of Depressive Symptomatology; MDD, major depressive disorder; MINI, Mini International Neuropsychiatric Interview; NR, not reported; OCD, obsessive-compulsive disorder; PD, panic disorder; PHQ, Patient Health Questionnaire; PST, problem-solving treatment; SC, stepped care; SCL-90-R, Symptom Checklist–90–Revised; SF-36, Short-Form Health Survey–36 items; SH, self-help; Tic-P, Trimbos/iMTA Questionnaire for Costs Associated with Psychiatric Illness; WSAS, Work and Social Adjustment Scale; Y-BOCS, Yale-Brown Obsessive-Compulsive Scale.

**Table 2:** Source of Treatment Content, Treatment Type, Recruitment Method, Attrition Rate, Reasons for Attrition, and Stepping Up Criteria.

No.	Study authors (year)	Treatment content	Recruitment	Cumulative attrition rate (step no./assessment)	Stepping up criteria (direct to the last step)
**Stepped Care Prevention**					
1	Apil *et al*.^30^,^31^	Step 2–SH CBT:Based on *Coping with Depression* (book)Step 3–FTF CBT:Individual *Coping with Depression* course	Psychiatric center	SC: 16.9% (PT)	CES-D >15 (diagnosis of depression by MINI)
2	Dozeman *et al*.^32^	Step 2–SH activity-scheduling:Based on *Coping with Depression* (book)Step 3–FTF life review:Based on *Life Review Therapy Using Autobiographical retrieval Practice for Older Adults with Depressive* Symptomatology	Residential homes	SC: 18.9% (PT)CAU: 10.8% (PT)	CES-D <5 improvement (DSM-IV diagnosis of depressive or anxiety disorder)
3	van’t Veer-Tazelaar *et al*.^33^,^34^	Step 2–SH CBT:Based on *Coping with Depression* (book)Step 3–FTF PST:A brief CBT that focuses on practical skill building and help regaining control of patients’ lives.	Primary care	SC: 11.8% (6 mo-PT), 14.7% (12 mo-FU), 15.3% (18 mo-FU), 15.9% (24 mo-FU)CAU: 4.1% (6 mo-PT), 4.7% (12 mo-FU), 5.9% (18 mo-FU), 8.2% (24 mo-FU)	CES-D ≥16 (diagnosis of depressive or anxiety disorders by MINI)
4	Zhang *et al*.^35^	Step 2–Guided SH instruction through telephone:Based on *Theory and Practice of Counseling and Psychotherapy, Theories in Counseling and Therapy: An Experiential Approach* and *Assessing Families and Couples: From Symptom to System* (books)Step 3–FTF PST:Based on *Problem-solving therapy: A social competence approach to clinical intervention* and *Problem Solving Therapy*	Primary clinics	SC: 6.6% (3 mo), 10.7% (6 mo), 10.7% (9 mo), 12.4% (12 mo-PT), 14.0% (15 mo-FU)CAU: 6.7% (3 mo), 8.4% (6 mo), 10.9% (9 mo), 11.8% (12 mo-PT), 15.1% (15 mo-FU)	CES-D ≥16 or HADS-A ≥6, without the SCID diagnosed MDD or GAD (NR)
**Stepped Care Treatment**					
5	Muntingh *et al*.^16^	Step 1–Guided SH CBT:Provided with psychoeducation, cognitive behavioural exercises and a guided relaxation CD (book) +consultationStep 2–FTF CBT:Cognitive therapy and exposure+workbook	Primary care	NR	BAI >11 or BAI <50% score reduction (NR)
6	Oosterbaan *et al*.^36^	Step 1–Guided SH CBT:Depression: Based on *Coping with Depression* Anxiety: Based on *Stresspac* (book)	Mental health care center	SC: 8.9% (PT), 16.5% (FU)CAU: 3.2% (PT), 7.6% (FU)	CGI-S ≥3 (depression with psychotic features, actively suicidal or family of the patient was overly strained due to psychiatric disorder)
7	Seekles *et al*.^18^	Step 2–Guided SH CBT:PST (book/Internet)/Exposure therapy for phobia (book) +feedbackStep 3–FTF PST:Based on Problem Solving Treatment for Anxiety and Depression: A Practical Guide	Mental health centers	SC: 29.2% (PT)CAU: NR	IDS ≥14, HADS ≥8 and WSAS ≥6 (WSAS ≥8 on 3 of the 4 daily functioning domains)
8	Tolin *et al*.^19^	Step 1–Guided SH ERP:Based on *Stop Obsessing!: How to Overcome Your Obsessions and Compulsions* (book)Step 2–FTF ERP:Based on Mastery of Obsessive-Compulsive Disorder: A Cognitive-Behavioral Approach (Therapist Guide)	NR	SC: 20.0% (PT)CAU: 6.7% (PT)	Y-BOCS >13 (Y-BOCS >13)

BAI, Beck Anxiety Inventory; CAU, care-as-usual; CBT, cognitive-behavioral therapy; CES-D, Centre of Epidemiological Studies–Depression scale; CGI-S, Clinical Global Impression–Severity Scale; DSM-IV, Diagnostic and Statistical Manual of Mental Disorders, Fourth Edition; FTF, face-to-face; FU, follow-up; GAD, generalized anxiety disorder; HADS, Hospital Anxiety and Depression Scale; IDS, Inventory of Depressive Symptomatology; MDD, major depressive disorder; MINI, Mini International Neuropsychiatric Interview; NR, not reported; PST, problem-solving treatment; PT, posttreatment; SC, stepped care; SCID, Structured Clinical Interview for DSM-IV; SH, self-help; WSAS, Work and Social Adjustment Scale; Y-BOCS, Yale-Brown Obsessive-Compulsive Scale.
