# Supplementary material for: The Efficacy and Cost-Effectiveness of Stepped Care Prevention and Treatment for Depressive and/or Anxiety Disorders: A Systematic Review and Meta-Analysis
Source: Sci Rep. 2016 Jul 5;6:29281. doi: 10.1038/srep29281 (PMC4932532; doi:10.1038/srep29281)
Supplement: Supplementary Information [file srep29281-s1.doc]

The Efficacy and Cost-Effectiveness of Stepped Care Prevention and Treatment for Depressive and/or Anxiety Disorders: A Systematic Review and Meta-Analysis

Fiona Yan-Yee Ho^a^, MPhil; Wing-Fai Yeung^b^, PhD; Tommy Ho-Yee Ng^c^, MPhil;
Christian S. Chan^a*^, PhD

^a^Department of Psychology, The University of Hong Kong, Hong Kong

^b^School of Nursing, The Hong Kong Polytechnic University, Hong Kong

^c^Department of Psychology, Temple University, United States

^*^Correspondence. Christian S. Chan, Department of Psychology, The University of Hong Kong, Pokfulam Road, Hong Kong. Telephone: +852 3917 7121. Fax: +852 2858 3518. E-mail: shaunlyn@hku.hk

**Supplementary Figure S1.** An Evidence-Based Stepped Care Model^9^

**
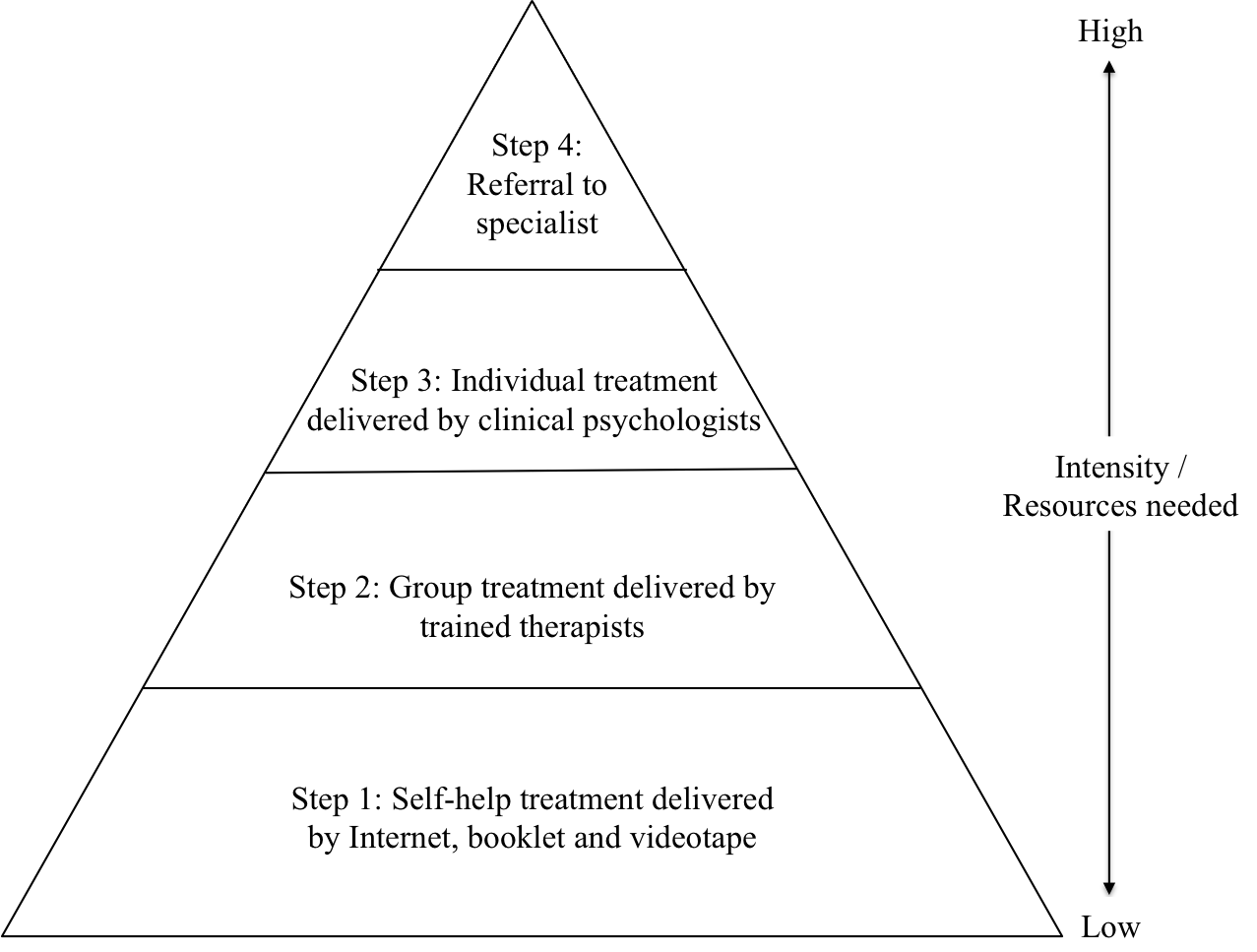
**

**Supplementary Table S1.** Major Inclusion and Exclusion Criteria

AUDIT, Alcohol Use Disorders Identification Test; CES-D, Centre of Epidemiological Studies – Depression scale; CGI, Clinical Global Impression; DSM-IV, Diagnostic and Statistical Manual of Mental Disorders, Fourth Edition; HADS, Hospital Anxiety and Depression Scale; Y-BOCS, Yale-Brown Obsessive-Compulsive Scale

| No. | Study authors (year) | Major inclusion and exclusion criteria |
| --- | --- | --- |
| **Stepped Care Prevention** | | |
| 1 | Apil et al. (2012, 2014) | Aged ≥ 55 y and previous psychological and/or medical treatment for depression. Absence of current psychotherapy for major depressive disorder, psychotic disorder, dysthymic disorder, bipolar disorder, primary anxiety disorder, or substance abuse or dependence. |
| 2 | Dozeman et al. (2012) | Elderly in residential homes, CES-D ≥ 8. Absence of DSM-IV depressive or anxiety disorder and severe cognitive impairment. |
| 3 | van’t Veer-Tazelaar et al. (2009, 2011) | Aged ≥ 75 y and CES-D ≥ 16. Absence of DSM-IV criteria of depressive or anxiety disorder (panic disorder, agoraphobia, social phobia, or generalized anxiety disorder) in the past 12 mo and serious cognitive decline. |
| 4 | Zhang et al. (2014) | Aged ≥ 18 y, and CES-D ≥ 16 or HADS-A ≥ 6. Absence of DSM-IV criteria of major depression and/or anxiety disorders. |
| **Stepped Care Treatment** | | |
| 5 | Muntingh et al. (2014) | Primary care patients aged ≥ 18 y, DSM-IV criteria of panic disorder (with or without agoraphobia) and/or generalized anxiety disorder. Absence of suicidal ideas, dementia or other severe cognitive disorders, psychotic disorder, bipolar disorder, alcohol or drug dependence, unstable severe medical condition and current psychiatric or psychological treatment (> 2 contacts per mo). |
| 6 | Oosterbaan et al. (2013) | Outpatients aged ≥ 18 y and DSM-IV criteria of panic disorder, agoraphobia, social phobia, specific phobia, generalized anxiety disorder, unipolar major and minor depressive disorder, dysthymia, stress-related adjustment disorders or comorbid with posttraumatic stress disorder or obsessive-compulsive disorder. Absence of alcohol and drug dependence, dementia, psychotic disorder or bipolar disorder. |
| 7 | Seekles et al. (2011) | Aged 18-65 y, DSM-IV criteria of major depression (single episode or recurrent), dysthymia, panic disorder (with or without agoraphobia), social phobia and/or generalized anxiety disorder, minor depression (2-4 out of 9 DSM-IV symptoms and at least 1 had to be a core symptom), or minor anxiety (HADS ≥ 12 and dysfunctioning in daily life). Absence of psychotic or bipolar disorder, current (< 2 mo) medical or psychological treatment for psychological problems, prominent suicidal ideation, or AUDIT > 20. |
| 8 | Tolin et al. (2011) | Aged ≥ 18 y, DSM-IV criteria of obsessive-compulsive disorder ≥ 1 y (Y-BOCS ≥ 16 and CGI ≥ 4), and current stable psychiatric treatment ≥ 1 mo. Absence of concurrent symptoms or diagnosis that required immediate attention and/or interfere with treatment engagement. |

| No. | Study authors (year) | Random-sequence generation | Allocation concealment | Blinding of participants | Blinding of assessors | Incomplete outcome data addressed | Free of selective reporting | Free of other bias |
| --- | --- | --- | --- | --- | --- | --- | --- | --- |
| **Stepped Care Prevention** | | | | | | | | |
| 1 | Apil et al. (2012, 2014) | ? | ? | - | ? | + | + | + |
| 2 | Dozeman et al. (2012) | + | ? | - | + | + | + | + |
| 3 | van’t Veer-Tazelaar et al. (2009, 2011) | + | ? | - | + | + | + | + |
| 4 | Zhang et al. (2014) | + | ? | - | + | + | + | + |
| **Stepped Care Treatment** | | | | | | | | |
| 5 | Muntingh et al. (2014) | + | ? | - | + | ? | + | + |
| 6 | Oosterbaan et al. (2013) | + | ? | - | + | + | + | + |
| 7 | Seekles et al. (2011) | + | + | - | + | + | + | + |
| 8 | Tolin et al. (2011) | ? | ? | - | + | + | + | + |

**Supplementary Table S2.** Cochrane’s Risks of Bias Assessment of Stepped Care Treatment and Prevention for Depression and/or Anxiety

+, yes (low risk of bias); -, no (high risk of bias); ?, unclear (uncertain risk)

**Supplementary Figure S2.** Stepped Care Treatment vs. CAU control on the Treatment Response of Anxiety Disorders at Immediate Posttreatment.

| Study authors (year) | Weight | Odds ratio | 95% CI | Odds ratio, random, 95% CI |
| --- | --- | --- | --- | --- |
| Muntingh et al. (2014) | 73.7% | 2.40 | 1.14, 5.08 | 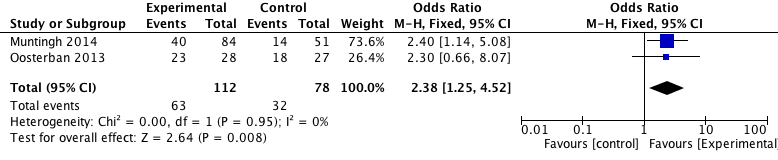  0.01 0.1 1 10 100  Favours control Favours experimental |
| Oosterbaan et al. (2013) | 26.3% | 2.30 | 0.66, 8.07 |
|  |  |  |  |
| Total (95% CI) | 100.0% | 2.38 | 1.25, 4.52 |
| Test for heterogeneity:  *χ^2^* = 0.00, *df* = 1 (*p* = .95), *I^2^* = 0%  Test for overall effect: *Z* = 2.64 (*p* = .008) | | | |
